# Potential clinical benefits of probiotics in pediatric allergic rhinitis: a systematic review and network meta-analysis

**DOI:** 10.3389/fped.2026.1744817

**Published:** 2026-03-09

**Authors:** Haiyan Li, Zeyu Chen, Lingyue Guo, Deng Liu, Dongmei Li, Xiaodong Jia, Keqiang Yan

**Affiliations:** 1 Tianjin Kingmed Diagnostics Laboratory Co. Ltd, Tianjin, China; 2 Tianjin Key Laboratory of Multi-omics Precision Diagnosis Technology for Neurological Diseases, Tianjin, China; 3 Tianjin University of Traditional Chinese Medicine, Tianjin, China; 4 Tianjin Medical University, Tianjin, China

**Keywords:** allergic rhinitis, children, meta-analysis, network meta-analysis, probiotics

## Abstract

**Objective:**

This study aimed to evaluate potential clinical benefits and superior strains of probiotics for pediatric allergic rhinitis (AR) using meta-analysis and network meta-analysis.

**Methods:**

A systematic search was conducted in databases including PubMed, Web of Science, Cochrane Library, and China National Knowledge Infrastructure up to July 31, 2025, to identify randomized controlled trials (RCTs). Inclusion criteria were pediatric patients with AR, probiotic interventions, control groups receiving placebo or standard treatment, and reported outcomes such as Total Nasal Symptom Score (TNSS), Pediatric Rhinoconjunctivitis Quality of Life Questionnaire (PRQLQ), Total Symptom Score (TSS), serum total IgE levels, or clinical efficacy. Study quality was assessed using the JADAD scale, with meta-analysis and network meta-analysis (NMA) performed via RevMan and R software, calculating standardized mean differences (SMD), relative risks (RR), and surface under the cumulative ranking curve (SUCRA) values.

**Results:**

Eighteen RCTs were included, involving 1,789 patients (963 in the probiotic group and 826 in the control group). Meta-analysis showed that probiotics significantly reduced TNSS (SMD = −0.85, 95%CI [−1.25, −0.44], *P* < 0.05), improved PRQLQ scores (SMD = −2.53, 95% CI [−4.66, −0.40], *P* < 0.05) and enhanced clinical efficacy (RR = 1.16, 95%CI [1.07, 1.25], *P* < 0.05). However, no significant effects were observed on TSS (SMD = −1.79, 95%CI: [−4.06, 0.48], *P* = 0.12), or serum total IgE levels (SMD = −0.34, 95%CI [−0.84, 0.16], *P* = 0.18). Subgroup and NMA analyses indicated that mixed strains performed superiorly across multiple outcomes.

**Conclusions:**

Probiotic supplementation, especially multi-strain formulations, may provide adjunctive benefits in pediatric AR, with potential for ameliorating nasal symptoms and enhancing quality of life, though further validation through rigorously designed trials is warranted.

## 1 Introduction

Allergic rhinitis (AR) is a common IgE-mediated inflammatory disorder characterized by nasal itching, congestion, rhinorrhea, and sneezing. Globally, AR affects hundreds of millions of individuals, imposing a substantial economic burden, including medical costs, absenteeism from work or school, and lost productivity. Children are particularly susceptible, with global prevalence rates ranging from 10% to 40% based on epidemiological surveys. The overall physician-diagnosed prevalence is 10.48%, while the self-reported current (within the past 12 months) prevalence stands at 18.12% (1). This elevated prevalence significantly impairs children's academic performance, quality of life, and psychological well-being, and may progress to complications such as asthma, resulting in long-term respiratory issues (2). Early intervention is essential, as untreated pediatric AR can exacerbate the atopic march and increase the risk of allergic diseases in adulthood (3). Standard treatments include antihistamines, intranasal corticosteroids, and immunotherapy, remain first-line interventions for pediatric allergic rhinitis per current international guidelines. While these treatments demonstrate well-established efficacy, clinical implementation may face challenges related to adherence barriers and heterogeneous therapeutic responses across populations. Emerging evidence positions probiotic interventions as potential immunomodulatory adjuncts to conventional pharmacotherapy, particularly for sustaining long-term remission in Th2-skewed phenotypes (4). This beneficial effect is largely attributed to the ability of probiotics to restore gut microbiota dysbiosis, a factor increasingly implicated in the pathogenesis of allergic diseases. Gut dysbiosis disrupts immune homeostasis by favoring a Th2-dominant response, whereas probiotics promote a shift toward a more balanced Th1/Th2 immune profile, thereby attenuating allergic inflammation (5).

The gut-nose axis, as an integral component of the immune system, suggests that microbiome dysbiosis may promote Th2-type immune responses, contributing to the onset and progression of AR (6). Probiotics, as live microbial supplements, can alleviate allergic reactions by strengthening the intestinal barrier, balancing Th1/Th2 responses, and promoting regulatory T cell (Treg) activity (7). Several randomized controlled trials (RCTs) have shown that strains such as Lactobacillus spp. and Bifidobacterium spp. reduce AR symptoms, though results vary: some report improvements, while others find no effects (8). Previous meta-analyses have predominantly focused on adults or mixed populations and have not adequately addressed strain specificity (9). Systematic evidence pertaining to pediatric AR remains limited, with a notable absence of network meta-analysis (NMA) to compare the relative efficacy of different strains (11). This study employs systematic literature retrieval and meta-analysis to evaluate the potential clinical benefits and superior strains of probiotics and in pediatric AR, with the aim of providing evidentiary support for clinical practice.

## 2 Materials and methods

### 2.1 Literature search strategy

A computerized literature search was conducted to identify RCTs investigating probiotic interventions for pediatric allergic rhinitis, spanning from database inception to July 31, 2025. The search strategy utilized a combination of subject headings and free-text terms, including the following key terms in Chinese and English: “过敏性鼻炎” (allergic rhinitis), “益生菌” (probiotics), “儿童” (children), “probiotics”, “allergic rhinitis” and “children”. Databases searched encompassed Chinese resources including China National Knowledge Infrastructure (CNKI), Wanfang Database, China Science and Technology Journal Database, China Biology Medicine disc (CBMdisc), and National Science and Technology Library (NSTL) and international platforms incorporating Web of Science, PubMed, Embase, and Cochrane Library. Additional studies were identified through manual screening of reference lists from included articles to ensure comprehensive coverage.

### 2.2 Literature inclusion and exclusion criteria

Inclusion criteria: 1) Study design: RCTs investigating probiotic interventions for AR, including parallel or crossover designs, regardless of blinding status, published in Chinese or English. 2) Participants: Pediatric patients diagnosed with AR. 3) Intervention: Probiotic formulations (e.g., Lactobacillus, Bifidobacterium, Saccharomyces, or Mixed-strain) administered via any route (oral, nasal, etc.), with no restrictions on dosage, administration route, or treatment duration. 4) Control groups: Placebo with identical dosage, standard therapy (e.g., antihistamines, intranasal corticosteroids), or no intervention. 5) Outcomes: At least one primary or secondary outcome reported. Primary outcomes included Total Nasal Symptom Score (TNSS), Pediatric Rhinoconjunctivitis Quality of Life Questionnaire (PRQLQ), Total Symptom Score (TSS), clinical efficacy and adverse outcomes. Secondary outcomes encompassed immunological parameters, such as serum total IgE levels before and after treatment.

Exclusion criteria: 1) Non-RCT studies, including case reports, case series, or retrospective studies. 2) Studies lacking a control group or unreported outcome data. 3) Studies focusing on non-allergic rhinitis (e.g., infectious rhinitis) or non-nasal allergic conditions. 4) Conference abstracts or studies with inaccessible full texts.

### 2.3 Selection of studies and data extraction

Following duplicate removal using EndNote 20 software, two independent researchers rigorously screened and extracted data in accordance with predefined inclusion and exclusion criteria. The results were cross-verified, with discrepancies resolved through third-party adjudication. Extracted data were recorded in an Excel spreadsheet, encompassing the following domains: 1) Study characteristics: First author, publication year, and country of origin. 2) Study design: Trial type (e.g., parallel, crossover) and blinding methodology (if applicable). 3) Participant details: Sample size and age range of enrolled subjects. 4) Intervention parameters: Probiotic strain(s), dosage regimen, and treatment duration. 5) Outcomes.

### 2.4 Quality assessment of the included studies

The methodological rigor of included studies was evaluated using the Jadad Scale (12), which assesses the following domains: 1) Random sequence generation. 2) Allocation concealment. 3) Blinding procedures. 4) Documentation of withdrawals and dropouts. Each criterion was scored independently, with the cumulative score representing the overall quality assessment outcome. Studies achieving a Jadad score ≥3 were classified as high-quality literature. Additionally, the Cochrane Risk of Bias Tool (13) was employed by two independent researchers to evaluate the risk of bias across seven domains: random sequence generation, allocation concealment, blinding of participants and personnel, blinding of outcome assessment, incomplete outcome data, selective reporting, and other potential biases. Each domain was rated as low, unclear or high risk of bias, and the overall risk of bias for individual studies was determined through a synthesis of domain-specific evaluations.

### 2.5 Statistical analysis

Meta-analyses were performed using Review Manager 5.3.0 (RevMan) and R 4.5.1 software. Heterogeneity was assessed via the I^2^ statistic, with I^2^ > 50% indicating substantial heterogeneity and warranting a random-effects model, while I^2^ ≤ 50% supported the use of a fixed-effects model. Subgroup analyses stratified by probiotic strains (e.g., Lactobacillus, Bifidobacterium) were conducted to explore potential sources of heterogeneity. NMA was further implemented to compare probiotics efficacy across strains, with ranking probabilities quantified using the Surface Under the Cumulative Ranking Curve (SUCRA). The SUCRA values ranges from 0 to 100%, with larger values indicating a higher ranking of the strain among all strains (13). Statistical significance was defined as *P* < 0.05.

## 3 Results

### 3.1 Literature screening results

**Figure 1:**
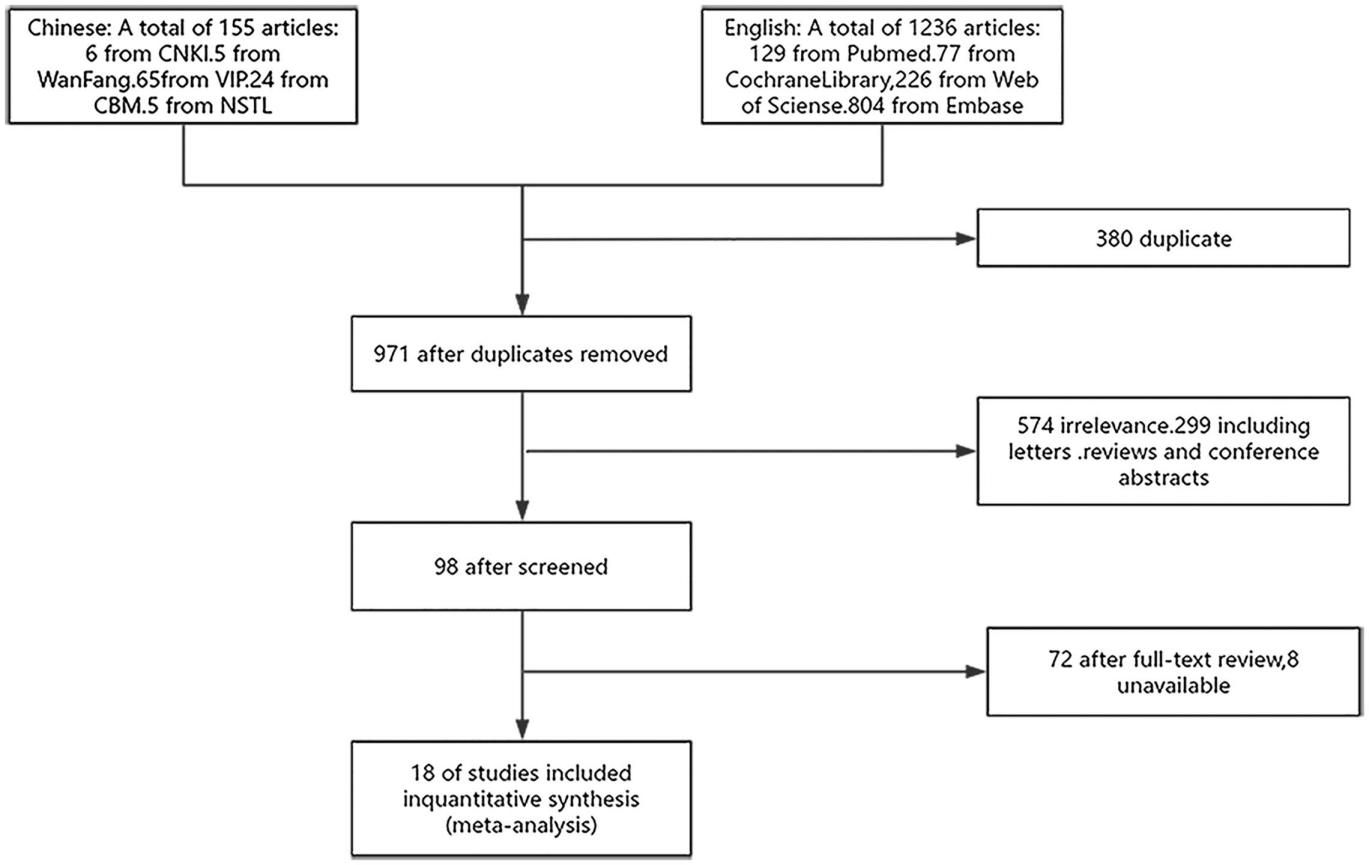
Flowchart of the search process for article included.

The initial search yielded 1,351 articles. After removing duplicates and screening titles and abstracts, 98 articles were retained. Full-text review and application of inclusion/exclusion criteria resulted in the final inclusion of 18 studies (Figure 1), comprising 4 Chinese-language and 14 English-language publications. All studies were RCTs, collectively involving 1,789 participants: 963 participants in probiotics intervention groups and 826 in control groups (placebo or standard therapy). Of the included studies, 50% (9/18) utilized probiotic monotherapy as the intervention, with the remaining 50% employing probiotic-standard therapy combinations, as detailed in Table 1. Key characteristics of the included studies are summarized in Table 2.

**Table 1:** Intervention types of studies.

Intervention Mode	N Studies	Sample Size
Probiotic monotherapy	9	962
Probiotic-standard therapy combinations	9	827

**Table 2:** Baseline characteristics of the studies included.

Author Year	Study Design	N	Age	Probiotics	Period	Control	Outcomes
Tao XY (14)	RCT	60	3–12y	Bifidobacterium, L. acidophilus and Enterococcus	8w	Antihistamines, Mometasone furoate nasal spray	Serum total IgE, Clinical efficacy
Zhai YN (15)	RCT	100	3–12y	Bifidobacterium longum, L.acidophilus and Enterococcus faecalis	8w	Glucocorticoids and *β*2-agonists	Clinical efficacy, total IgE
Qiu HY (16)	RCT	60	2–14y	Bifidobacterium infantis, L.acidophilus, Enterococcus faecalis, Bacillus cereus	3m	Flonase	Clinical efficacy
Rong JY (17)	RCT	128	6–14y	Bifidobacterium longum, Lactobacillus acidophilus and Enterococcus faecalis	6m	Conventional treatment: Environmental guidance, medication use based on condition, etc	Clinical efficacy, Serum total IgE
Ciprandi G (18)	RCT	20	12–15y	B.clausii	3w	Levocetirizine (5 mg tablet)	TSS, QoL, Nasal eosinophils
Michele Miraglia Del Giudice (19)	RCT-DB	40	9 ± 2.2y	B.longum BB536 (3 × 10^9 CFU), B.infantis M-63 (1 × 10^9 CFU), B.breve M-16 V (1 × 10^9 CFU)	8w	Placebo (Inert excipient), Cetirizine syrup	TSS, QoL
Nabil F (20)	RCT	30	5–18y	L. acidophilus	6m	SLIT (Date palm pollen + buffer solution)	TNSS
Lin TY (21)	RCT-DB	199	6–12y	L.salivarius	12w	Placebo (microcrystalline cellulose)	SSS, OSS, TNSS, total IgE
Peng GC (22)	RCT-DB	90	12–18y	L.paracasei	1m	Placebo (Milk powder)	QoL, PRQLQ
Jeong K (23)	RCT-DB	68	6–19y	Bifidobacterium longum and Lactobacillus plantarum (NVP-1703)	4w	Placebo (Maltodextrin)	Serum total IgE, TNSS, NSDS, QoL
Mubashir Ahmed (24)	RCT-DB	212	6–60m	L.paracasei	6w	Cetirizine	Clinical efficacy of rhinitis
Lin WY (25)	RCT-DB	60	6–13y	L.paracasei	12w	Levocetirizin; Placebo: Maltodextrin	TSS, TNSS, OSS
Lin EK (26	RCT-DB	122	5–16y	L.paracasei	3m	Placebo	Clinical symptom score, TNSS, Serum total IgE, IFN-*γ*
Jan RH (27)	RCT-DB	198	4–13y	L. rhamnosus	12w	Placebo (microcrystalline cellulose)	OSS, NSS, Hematological and total IgE
Qadir A (28)	RCT	160	3–60m	L.paracasei LP-33	6m	Cetirizine	Clinical symptom score, Treatment effective rate, Probability of adverse outcomes
Chen YS (29)	RCT-DB	105	6–12y	L.gasseri	10w	β2-agonist (Terbutaline aerosol), Oral corticosteroid (Prednisolone); Placebo (Starch)	Clinical symptom score, Improvement in AR symptoms, Serum total IgE
Wang MF (30)	RCT-DB	80	12–18y	L.paracasei LP-33	5w	Yogurt without LP-33 supplementation	PRQLQ
Lue KH (31)	RCT	63	7–12y	L. johnsonii EM1	24w	Levocetirizine	PRQLQ, NSS, TSS, Blood eosinophils

### 3.2 Quality assessment

**Figure 2:**
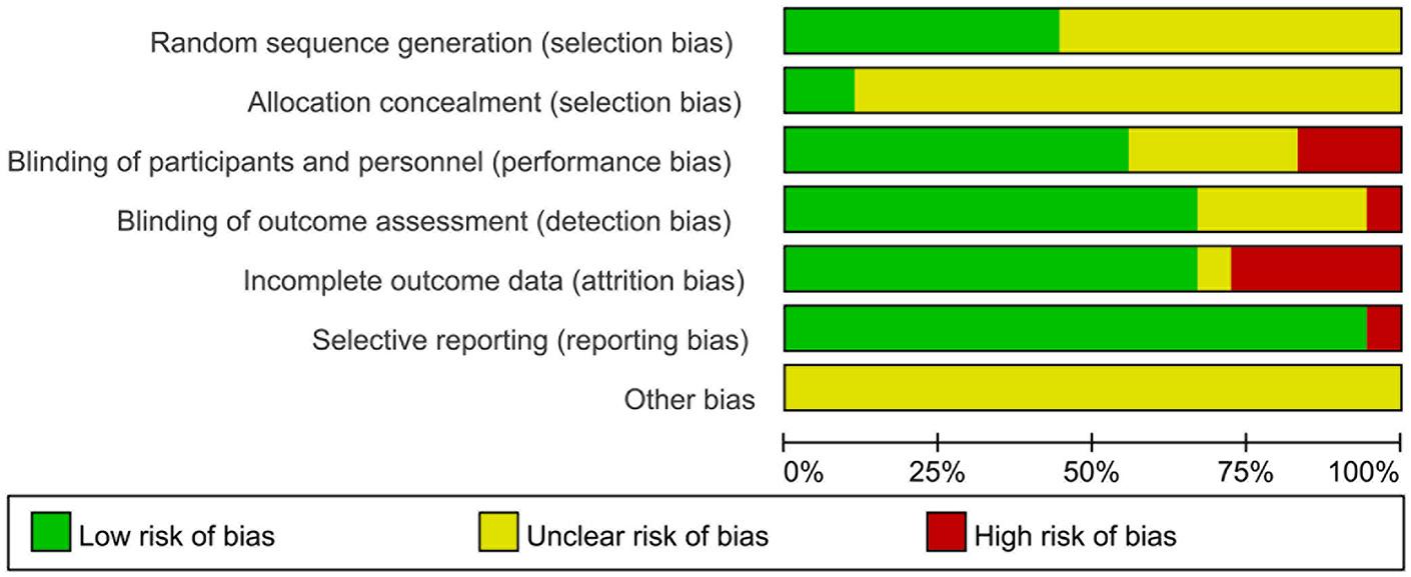
Risk of bias summary of included studies.

**Figure 3:**
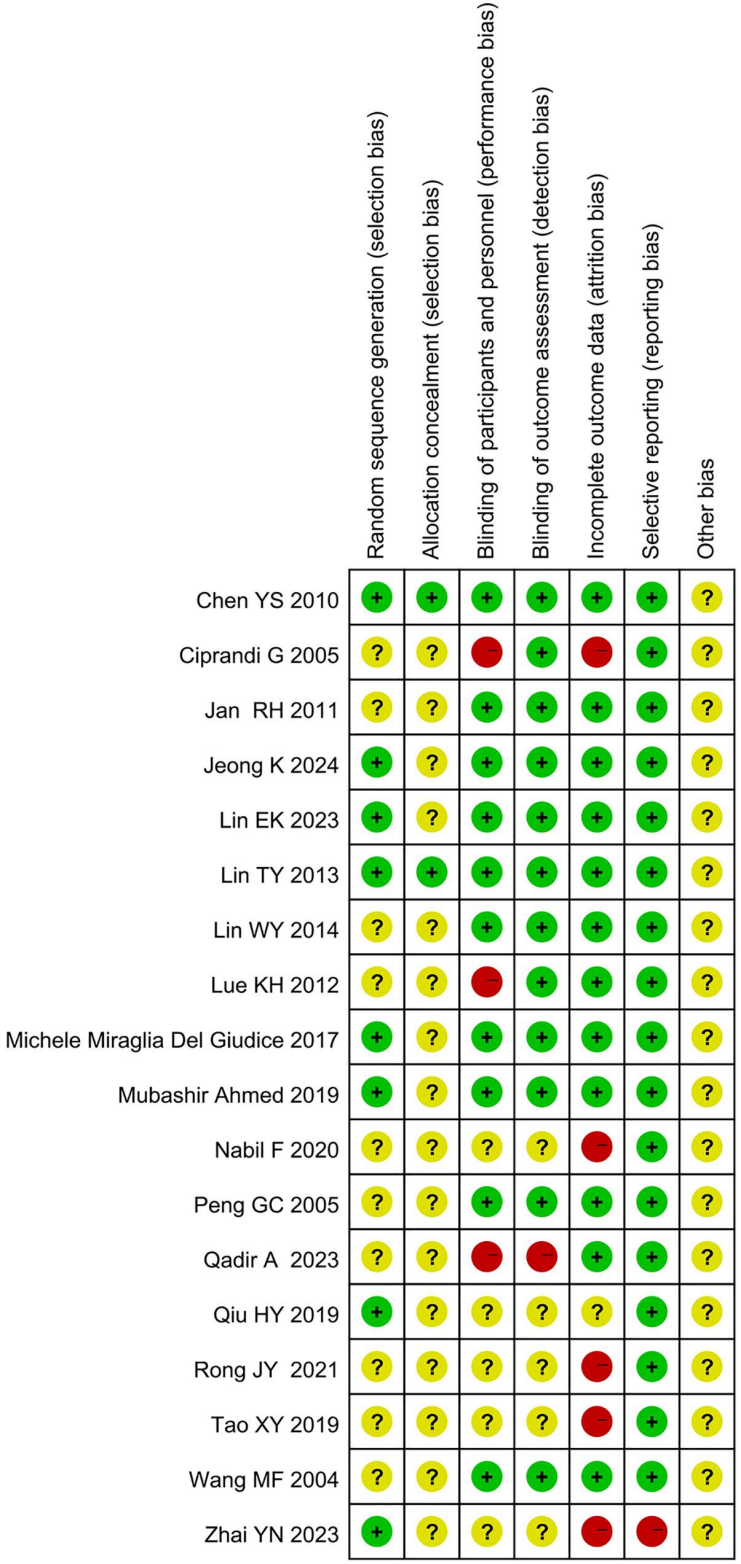
Risk of bias assessment for included studies.

Methodological quality evaluation using the Jadad scale identified 14 studies as high-quality literature (Table 3). Risk of bias assessment conducted via the Cochrane Risk of Bias Tool revealed 10 studies with low risk, 1 studies with unclear risk, and 7 studies with high risk of bias (summarized in Figure 2 and detailed in Figure 3).

**Table 3:** Jadad scale of the including studies.

Author	Randomization	Allocation concealment	Blinding	withdrawals and dropouts	Total Scores
Tao XY (14)	Unclear	Unclear	No	No	2
Zhai YN (15)	Yes	Unclear	No	No	3
Qiu HY (16)	Yes	Unclear	No	No	3
Rong JY (17)	Unclear	Unclear	No	No	2
Ciprandi G (18)	Unclear	Unclear	No	No	2
Michele Miraglia Del Giudice (19)	Yes	Unclear	Yes	Yes	6
Nabil F (20)	Unclear	Unclear	No	No	2
Lin TY (21)	Yes	Yes	Yes	Yes	7
Peng GC (22)	Unclear	Unclear	Yes	Yes	5
Jeong K (23)	Yes	Unclear	Yes	Yes	6
Mubashir Ahmed (24)	Yes	Unclear	Yes	Yes	6
Lin WY (25)	Unclear	Unclear	Yes	Yes	5
Lin EK (26)	Yes	Unclear	Yes	Yes	6
Jan RH (27)	Unclear	Unclear	Yes	Yes	5
Qadir A (28)	Unclear	Unclear	No	Yes	3
Chen YS (29)	Yes	Yes	Yes	Yes	7
Wang MF (30)	Unclear	Unclear	Yes	Yes	5
Lue KH (31)	Unclear	Unclear	No	Yes	3

### 3.3 Results of meta-analysis

#### 3.3.1 Effects of probiotics on TNSS

**Figure 4:**
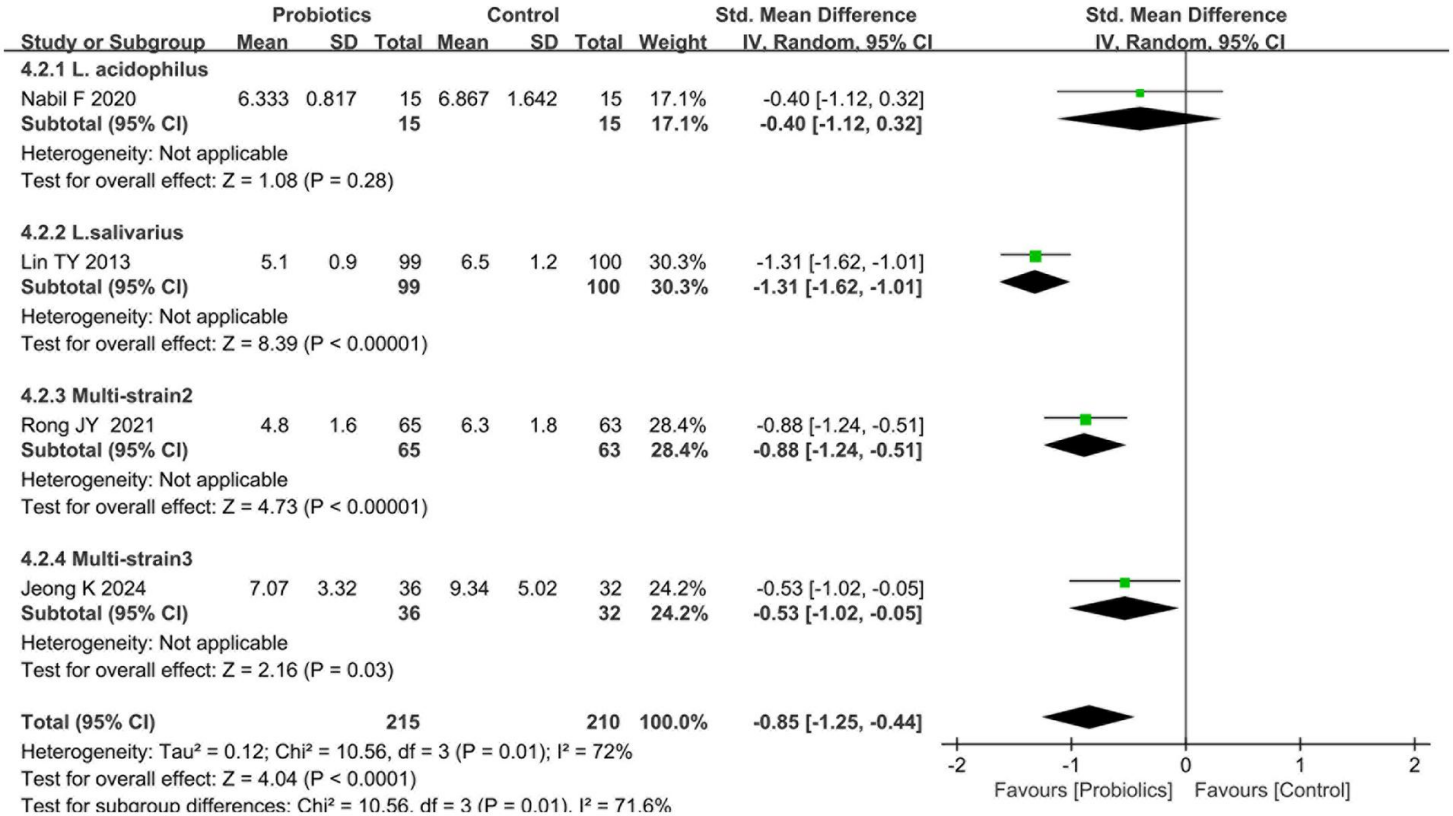
Forest plot comparing the effect of probiotics and control on TNSS. Subgroup by the types of probiotics.

Four studies (17, 20, 21, 23) reported post-treatment TNSS comparisons between probiotics and control groups. Heterogeneity testing revealed significant between-study variation (I^2^ = 72%, *P* = 0.01), prompting adoption of a random-effects model. The pooled analysis demonstrated statistically significant reductions in TNSS scores favoring probiotic interventions [standardized mean difference [SMD]  = −0.85, 95%CI [−1.25, −0.44], *P* < 0.0001], as illustrated in Figure 4.

Subgroup analyses were conducted according to the category of probiotics. Two studies (14, 26) evaluating multi-strain formulations (Multi-strain2: B. longum, L. acidophilus, and E. faecalis; Multi-strain3: B. longum and L. plantarum [NVP-1703]) both reported statistically significant TNSS difference between probiotics and controls (*P* < 0.05). In contrast, two studies (22, 24) assessing single-strain interventions showed divergent outcomes: L. acidophilus exhibited no significant TNSS improvement (*P* = 0.28), whereas L. salivarius demonstrated clinically meaningful reductions (*P* < 0.05; Figure 4). Additionally, subgroup analyses demonstrated significant reductions in TNSS scores for both probiotic-standard therapy combinations [vs. standard care alone: SMD = −0.75, 95%CI: (−1.16, −0.33), *P* = 0.01] and probiotic monotherapy [vs. placebo: SMD = −0.95, 95%CI: (−1.71, −0.18), *P* < 0.001], as detailed in Supplementary Figure S1.

#### 3.3.2 Effects of probiotics on serum total IgE

**Figure 5:**
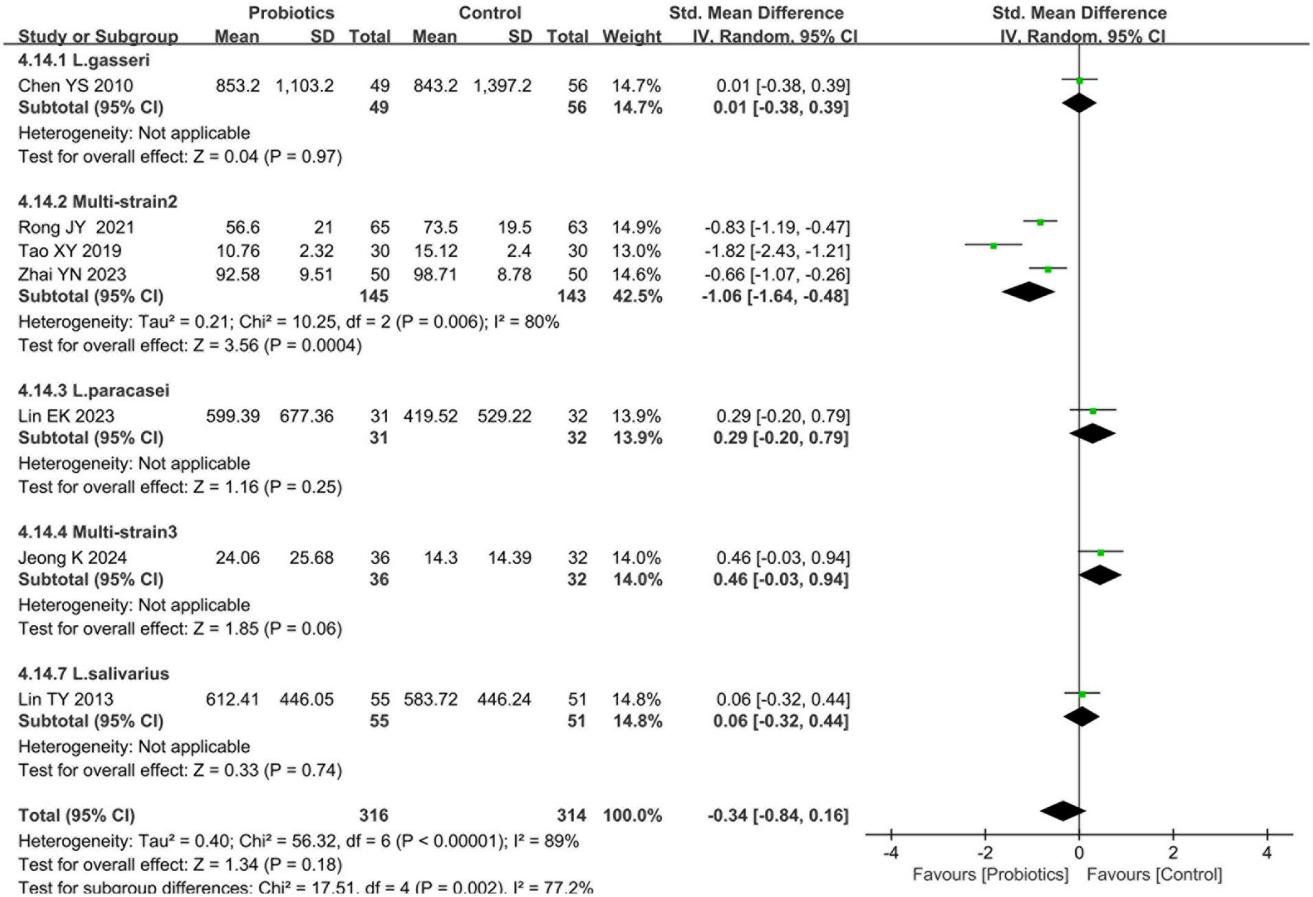
Forest plot comparing the effect of probiotics and control on the serum total IgE. Subgroup by the types of probiotics.

Seven studies (14, 15, 17, 23, 26, 29) reported serum total IgE levels following probiotics interventions compared to controls. Heterogeneity testing indicated substantial between-study variation (I^2^ = 89%, *P* < 0.0001), necessitating a random-effects model. Pooled analysis revealed no statistically significant difference in post-treatment serum total IgE level between probiotics and control groups [SMD = −0.34, 95%CI (−0.84, −0.16), *P* = 0.18], as shown in Figure 5.

Subgroup analyses stratified by probiotic strains demonstrated heterogeneous effects. Three studies (14, 15, 17) evaluating Multi-strain2 (B. longum, L. acidophilus, and E. faecalis) exhibited significant heterogeneity (I^2^ = 80%, *P* < 0.01), with a random-effects model showing statistically significant difference in serum total IgE levels between two groups [SMD = −1.06, 95%CI (−1.64, −0.48), *P* < 0.001]. In contrast, three additional studies (23, 26, 29) investigating Multi-strain3 (B. longum and L. plantarum [NVP-1703]), L. gasseri and L. paracasei interventions reported no statistically significant IgE modulation (*P* = 0.06, *P* = 0.97 and *P* = 0.25, respectively; Figure 5). In addition, subgroup analyses stratified by probiotic administration modality revealed that probiotic-standard therapy combinations (vs. standard care alone) significantly reduced serum total IgE levels [SMD = −1.06, 95%CI: (−1.64, −0.48) *P* < 0.001], whereas no significant difference was observed between probiotic monotherapy and placebo groups [SMD = −0.17, 95%CI: (−0.05, 0.38), *P* = 0.13], as illustrated in Supplementary Figure S2.

#### 3.3.3 Effects of probiotics on PRQLQ

**Figure 6:**
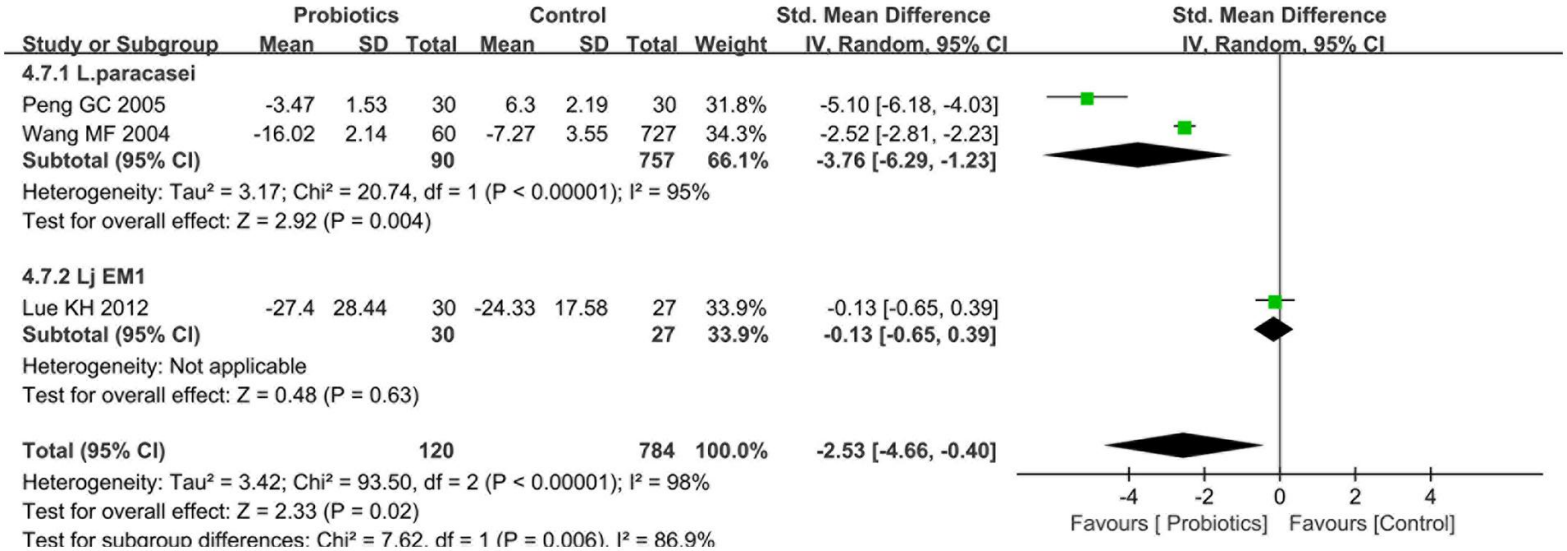
Forest plot comparing the effect of probiotics and control on PRQLQ.

Three studies (22, 30, 31) reported post-treatment PRQLQ scores between probiotics and control groups. Heterogeneity testing revealed significant between-study variation (I^2^ = 98%, *P* < 0.0001), warranting a random-effects model. The pooled analysis demonstrated no statistically significant improvement in PRQLQ scores favoring probiotic interventions [SMD = −2.53, 95%CI (−4.66, −0.40), *P* = 0.02], as illustrated in Figure 6.

Subgroup analyses stratified by probiotic strains demonstrated that Lactobacillus paracasei significantly improved PRQLQ scores compared to controls [SMD=−3.76, 95%CI: (−6.29, 1.23), *P* = 0.004], whereas no significant difference was observed for Lactobacillus johnsonii EM1 [Lj EM1, SMD = −0.13, 95%CI: (−0.65, 0.39), *P* = 0.62] (Figure 6). In parallel, subgroup analyses based on therapeutic modality revealed that probiotic monotherapy (vs. placebo) achieved significant PRQLQ score improvements [SMD = −3.76, 95%CI: (−6.29, 1.23), *P* = 0.004], whereas probiotic-standard therapy combinations (vs. standard care alone) showed no statistically significant effect [SMD = −0.13, 95%CI: (−0.65, 0.39), *P* = 0.62] (Supplementary Figure S3).

#### 3.3.4 Effects of probiotics on clinical efficacy

**Figure 7:**
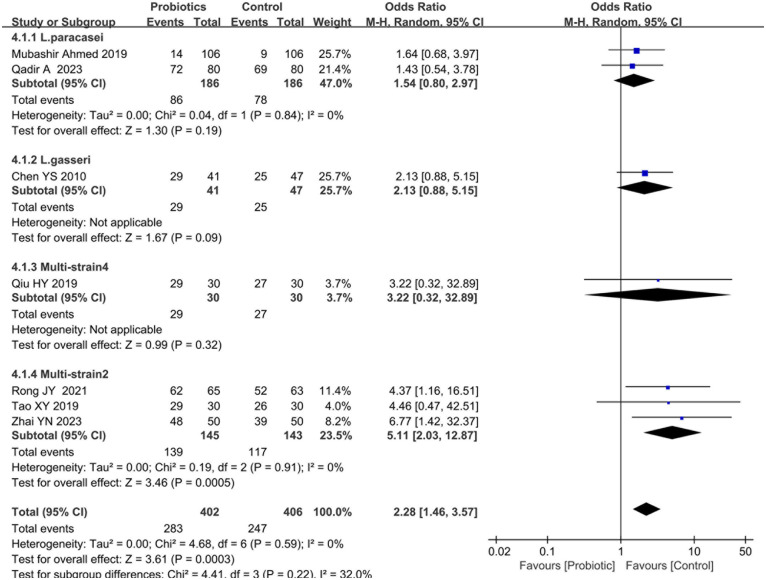
Forest plot comparing the effect of probiotics and control on clinical efficacy.

Seven studies (14–17, 24, 28, 29) evaluated the therapeutic efficacy of probiotic interventions compared to controls. Heterogeneity testing revealed no significant between-study variation (I^2^ = 0%, *P* = 0.59), supporting the use of a fixed-effects model. Pooled analysis demonstrated a statistically significant improvement in clinical efficacy favoring probiotic groups [risk ratio [RR] = 2.28, 95%CI [1.46, 3.57], *P* < 0.001], as detailed in Figure 7.

Subgroup analyses stratified by probiotic strains revealed no statistically significant differences in clinical efficacy between L. paracasei [SMD = 1.54, 95%CI: (0.80, 2.97), *P* = 0.19], L. gasseri [SMD = 2.13, 95%CI: (0.88, 5.15), *P* = 0.09], or Multi-strain4 [SMD = 3.22, 95%CI: (0.32, 32.89), *P* = 0.32] compared to control groups. However, Multi-strain2 exhibited significant therapeutic superiority [SMD = 5.11, 95%CI: (2.03, 12.87), *P* < 0.001, Figure 7]. In contrast, subgroup analyses based on therapeutic modality demonstrated that probiotic-standard therapy combinations (vs. standard care alone) significantly enhanced clinical efficacy [SMD = 2.48, 95%CI: (1.50, 4.10), *P* < 0.001], whereas probiotic monotherapy (vs. placebo) showed no statistically significant improvement [SMD=2.13, 95%CI: (0.88, 5.15), *P* = 0.09], as detailed in Supplementary Figure S4.

#### 3.3.5 Effects of probiotics on TSS

**Figure 8:**
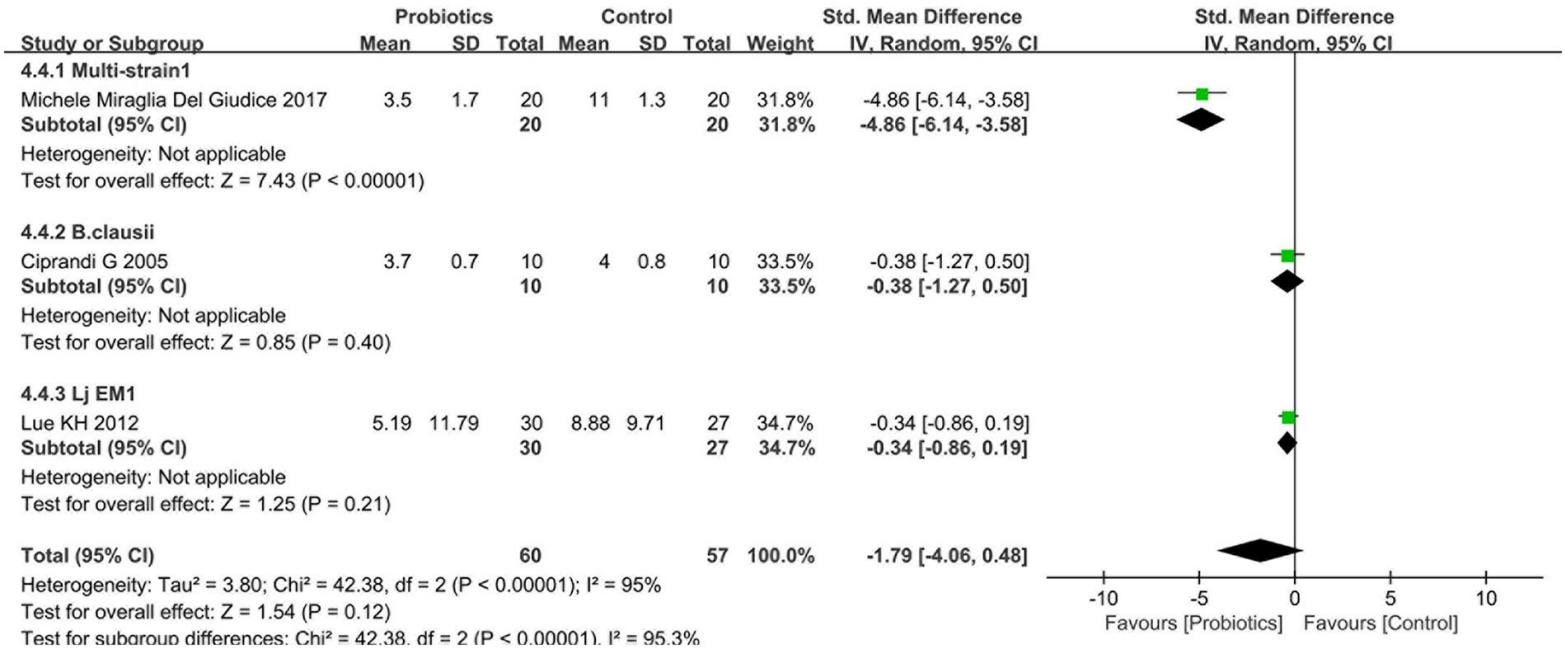
Forest plot comparing the effect of probiotics and control on TSS.

Three studies (18, 19, 31) investigated post-treatment TSS comparing probiotic and control groups. Heterogeneity testing indicated significant between-study variation (I^2^ = 95%, *P* < 0.0001), justifying the use of a random-effects model. Pooled analysis revealed no statistically significant differences between two groups [SMD = −1.79, 95%CI (−4.06, 0.48), *P* = 0.12], as illustrated in Figure 8.

Subgroup analyses stratified by probiotic strains revealed that Multi-strain1 significantly reduced TSS scores compared to controls [SMD = −4.86, 95%CI: (−6.14, −3.58), *P* < 0.001], whereas neither B. clausii [SMD = −0.38, 95%CI: (−1.27,0.50), *P* = 0.40] nor Lj EM1 [SMD = −0.34, 95%CI: (−0.86, 0.19), *P* = 0.21] demonstrated statistically significant differences in TSS scores (Figure 8). In contrast, subgroup analyses based on therapeutic modality indicated that probiotic monotherapy (vs. placebo) achieved significant reductions in TSS scores [SMD = −4.86, 95%CI: (−6.14, −3.58), *P* < 0.001], while probiotic-standard therapy combinations (vs. standard care alone) showed no statistically significant improvement [SMD=−0.35, 95%CI: (−0.80, 0.10), *P* = 0.12], as detailed in Supplementary Figure S5.

### 3.4 SUCRA analysis identified superior probiotic strains

In the NMA, network connectivity was ensured by requiring all interventions to be indirectly comparable through a shared comparator. To achieve this, all non-probiotic interventions were consolidated into a unified “Control” group within the NMA framework. This approach was predicated on the assumption that all studies were conducted under similar standard-of-care protocols, with probiotics serving as the sole variable. The consolidation aimed to evaluate the relative efficacy of distinct probiotic strains compared to conventional management strategies.

#### 3.4.1 Rank of diverse probiotic strains on TSS

**Figure 9:**
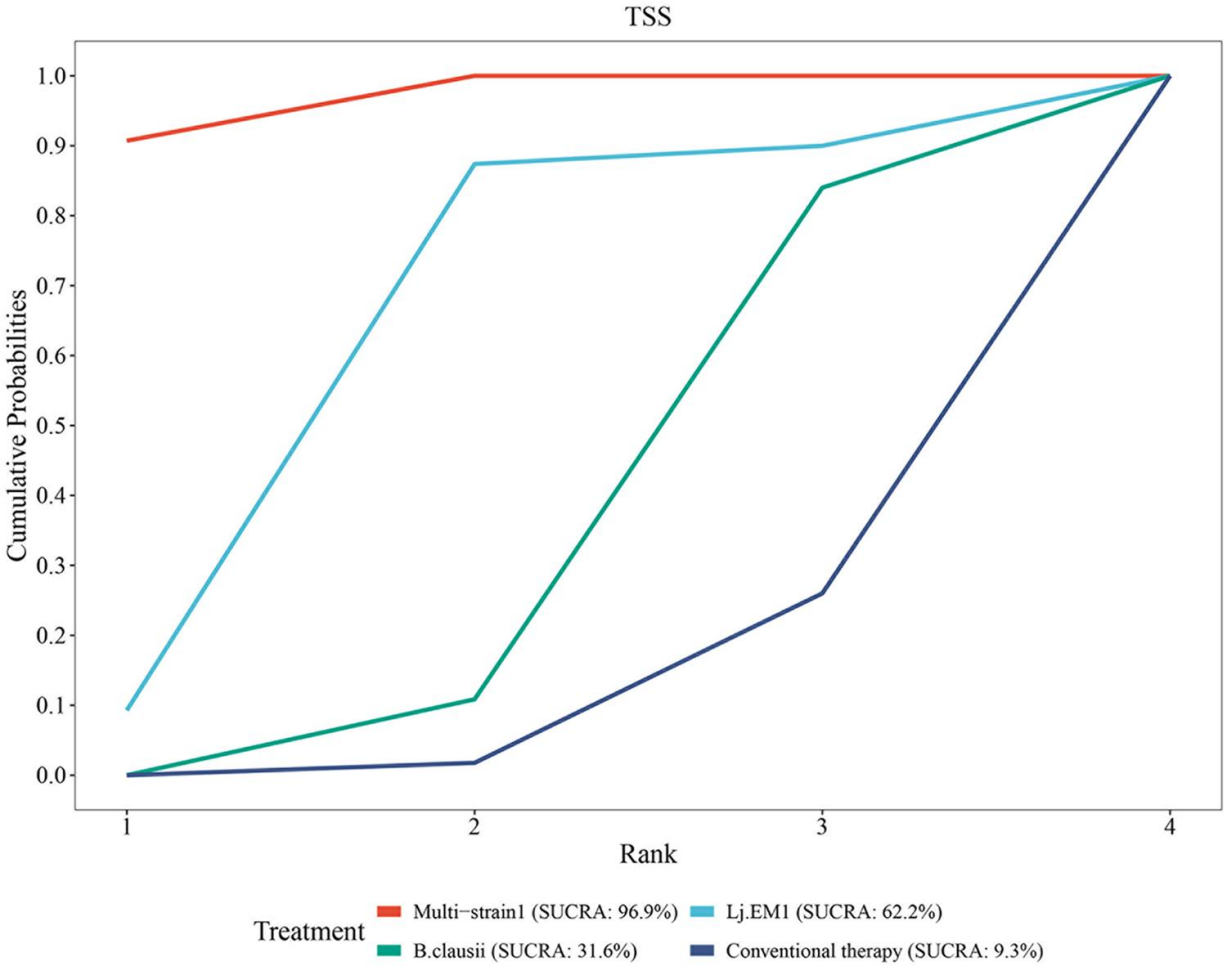
SUCRA probability rank of TSS.

SUCRA analysis revealed that Multi-strain1 (B. longum subsp. longum BB536, B. infantis M-63, and B. breve M-16), Lj EM1 and B. clausii demonstrated superior efficacy in reducing TSS compared to conventional therapy, with ranking probabilities as follows: Multi-strain1 (SUCRA = 96.9%) > Lj EM1 (SUCRA = 62.2%) > B. clausii (SUCRA = 31.6%) > conventional therapy (SUCRA = 9.3%) (Figure 9).

#### 3.4.2 Rank of diverse probiotic strains on clinical efficacy

**Figure 10:**
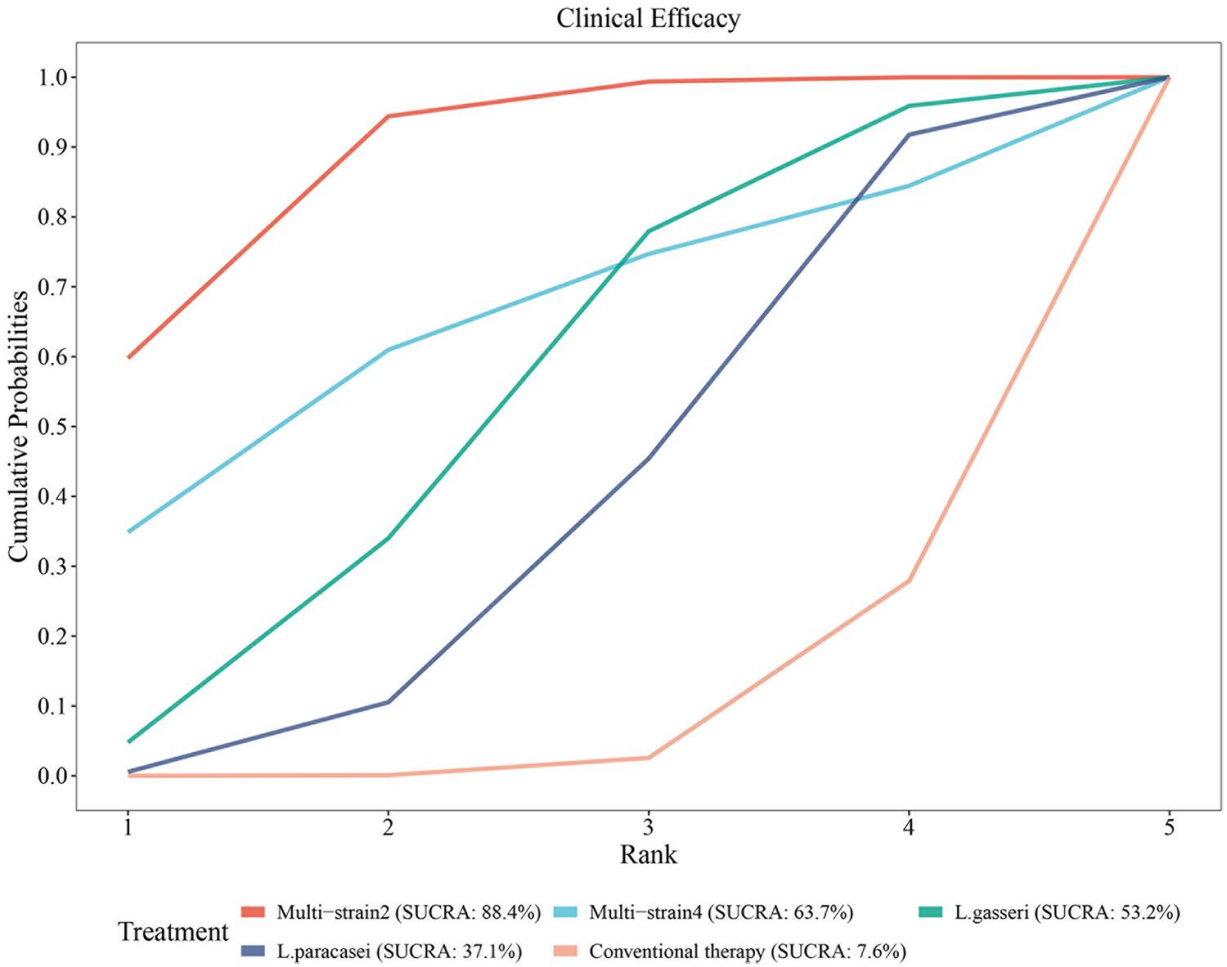
SUCRA probability rank of clinical efficacy.

SUCRA analysis demonstrated superior efficacy of multi-strain probiotics and specific Lactobacillus strains over conventional therapy. The ranking probabilities were as follows: Multi-strain2 (B. longum, L. acidophilus, and E. faecalis; SUCRA = 88.4%) > Multi-strain4 (B. infantis, L. acidophilus, E. faecalis, and B. cereus; SUCRA = 63.7%) > L. gasseri (SUCRA = 53.2%) > L. paracasei (SUCRA = 37.1%) > conventional therapy (SUCRA = 7.6%) (Figure 10).

#### 3.4.3 Rank of diverse probiotic strains on PRQLQ

**Figure 11:**
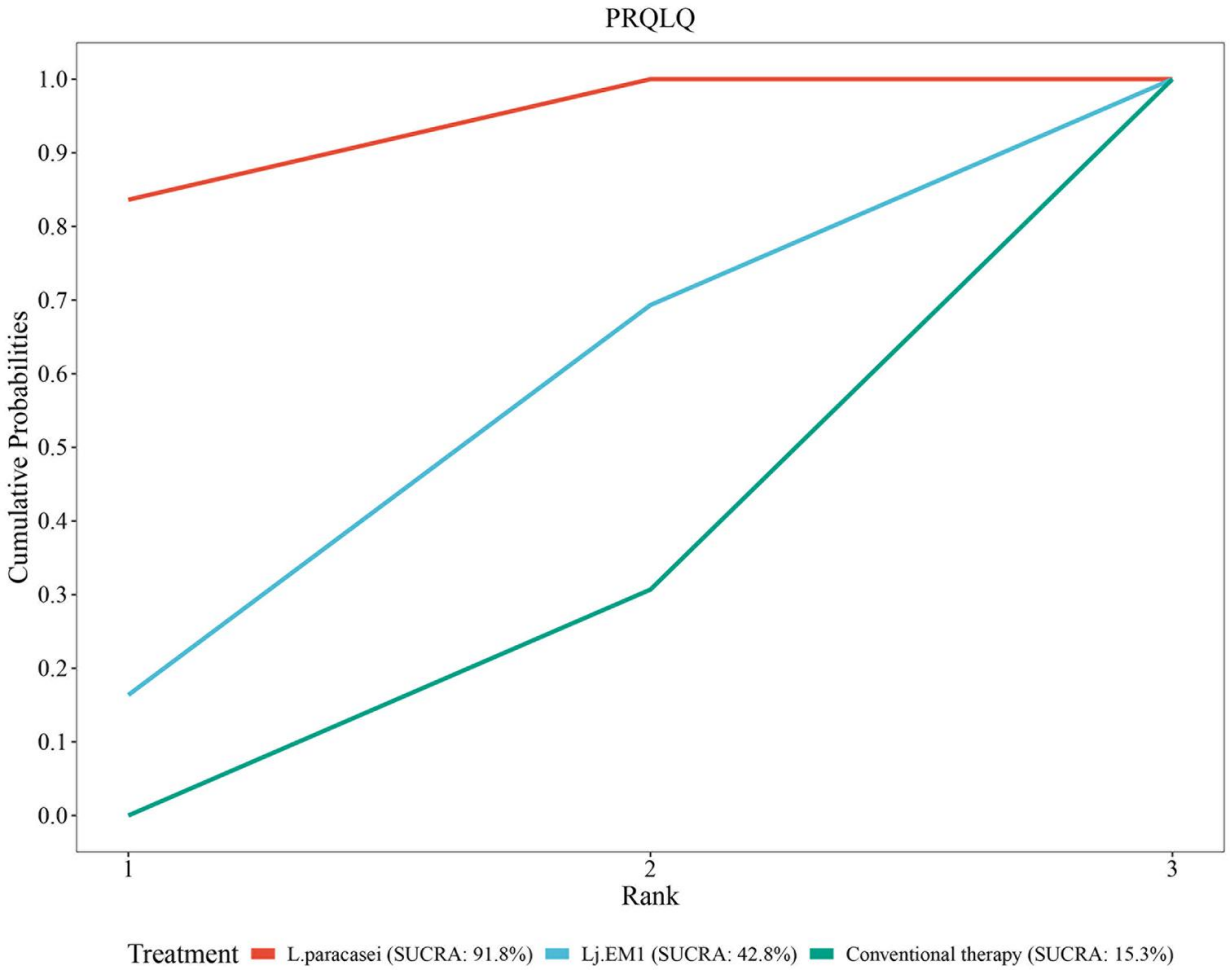
SUCRA probability rank of PRQLQ.

SUCRA analysis disclosed superior efficacy of L. paracasei and Lj EM1 compared to conventional therapy. The ranking probabilities were as follows: L. paracasei (SUCR = 91.8%) > Lj EM1 (SUCRA =  42.8%) > conventional therapy (SUCRA = 15.3%) (Figure 11).

#### 3.4.4 Rank of diverse probiotic strains on TNSS

**Figure 12:**
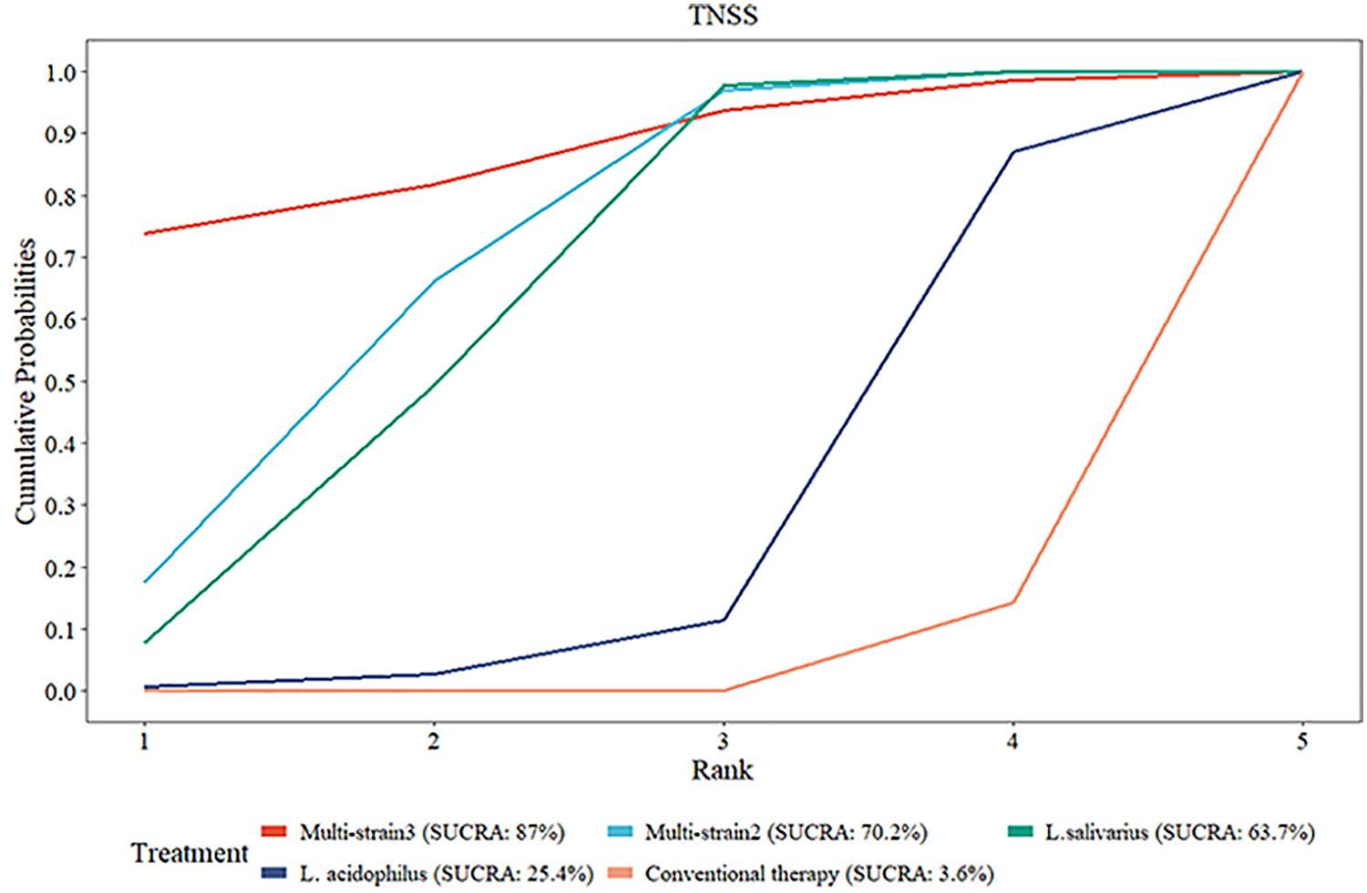
SUCRA probability rank of TNSS.

SUCRA analysis exposed superior efficacy of multi-strain probiotics and Lactobacillus strains in reducing TNSS compared to conventional therapy. The ranking probabilities were as follows: Multi-strain3 [B. longum and L. plantarum (NVP-1703); SUCRA = 87.0%] > Multi-strain2 (B. longum, L. acidophilus, and E. faecalis; SUCRA = 70.2%) > L. salivarius (SUCRA = 63.7%) > L. acidophilus (SUCRA = 25.4%) > conventional therapy (SUCRA = 3.6%) (Figure 12).

#### 3.4.5 Rank of diverse probiotic strains on serum total IgE

**Figure 13:**
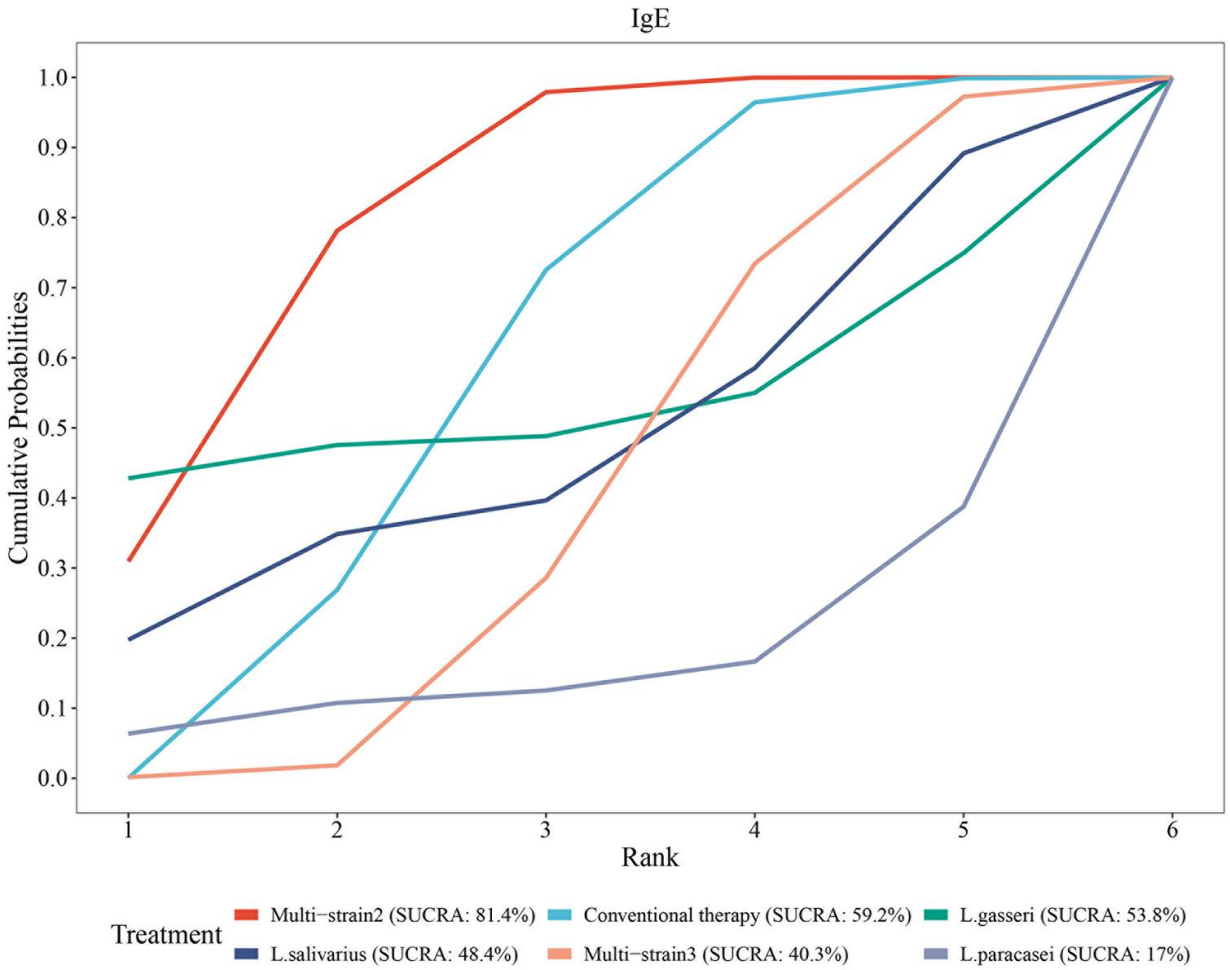
SUCRA probability rank of the serum total IgE.

SUCRA analysis revealed superior efficacy of multi-strain probiotics and L. gasseri in reducing serum total IgE levels compared to conventional therapy, while conventional therapy outperformed Multi-strain3, L. paracasei, L. salivarius and L. gasseri. The ranking probabilities were as follows: Multi-strain2 (B. longum, L. acidophilus, and E. faecalis; SUCRA = 81.4%) > conventional therapy (SUCRA = 59.2%) > L. gasseri (SUCRA = 53.8%) > L. salivarius (SUCRA = 48.4%) > Multi-strain3 [B. longum and L. plantarum (NVP-1703); SUCRA = 40.3%] > L. paracasei (SUCRA = 17.0%) (Figure 13).

## 4 Discussion

This meta-analysis incorporated 18 randomized controlled trials, encompassing a total of 1,789 pediatric patients with AR, to systematically evaluate the potential clinical benefits and superior strains of probiotics. The findings indicate that probiotic treatment significantly reduces nasal symptom scores, enhances rhinitis-related quality of life, and improves clinical efficacy, although it exerts no overall significant impact on serum total IgE levels and TSS. These results provide evidentiary support for probiotics as an adjunctive treatment for pediatric AR, aligning with existing literature while underscoring the importance of strain specificity and heterogeneity.

First, regarding the amelioration of nasal symptoms, this study revealed that the TNSS in the probiotic group was significantly lower than in the control group, consistent with outcomes from several prior meta-analyses. For instance, Zajac et al. (32) reported in a meta-analysis of adult and pediatric allergic rhinitis that probiotics effectively alleviate nasal symptoms, such as nasal congestion, itching, and sneezing. Subgroup analyses further demonstrated that mixed strains outperformed single strains in reducing TNSS, potentially attributable to synergistic effects that enhance immune modulation and intestinal barrier function. SUCRA analysis confirmed that Multi-strain3 (SUCRA = 87.0%) and Multi-strain2 (SUCRA = 70.2%) ranked highest in TNSS improvement, suggesting a preference for mixed probiotic formulations in clinical practice. However, single strains such as L. acidophilus exhibited non-significant effects (*P* = 0.28), which may relate to strain specificity, dosage, and treatment duration, necessitating further optimization.

Second, allergic rhinitis, as an IgE-mediated type I hypersensitivity reaction, is pathophysiologically linked to Th2 immune responses. Although total IgE levels exhibit limited correlation with individual symptom severity, they remain designated as secondary endpoints in international allergen immunotherapy (AIT) guidelines, primarily because total IgE reflects systemic sensitization burden and demonstrates reproducible reductions following effective therapeutic interventions. This analysis showed no overall significant reduction in serum total IgE levels with probiotics, although certain mixed strains demonstrated notable differences in subgroup analyses. SUCRA analysis indicated that Multi-strain2 (SUCRA = 81.4%) performed best in reducing total IgE levels, whereas L. paracasei ranked poorly (SUCRA = 17.0%), emphasizing the critical role of strain selection. Probiotics may exert their effects through regulation of the Th1/Th2 balance, promotion of regulatory T cell (Treg) activity, and reduction of inflammatory cytokines; however, their modulation of serum total IgE is not universally applicable and should be evaluated in conjunction with individual immune status. Future investigations should adopt multidimensional assessments incorporating allergen-specific IgE profiles, cytokine profiles, and validated clinical symptom scales to comprehensively evaluate therapeutic outcomes.

In terms of quality of life and clinical efficacy, this study confirmed that probiotics significantly improve the total PRQLQ score and clinical outcomes, echoing the systematic review by Güvenç et al. (33), which demonstrated enhanced quality of life in patients with allergic rhinitis. Notably, subgroup analysis stratified probiotic monotherapy revealed nonsignificant clinical efficacy improvement, whereas probiotic-standard therapy combinations demonstrated enhanced clinical efficacy, suggesting potential adjunctive clinical value of probiotics. SUCRA analysis highlighted the superiority of Multi-strain2 (SUCRA = 88.4%) in clinical efficacy, suggesting that mixed strains may indirectly alleviate rhinitis symptoms by improving gut microbiome diversity. Additionally, although no significant difference in TSS was observed between probiotic and control groups overall, subgroup analyses revealed that Multi-strain1 significantly reduced TSS scores, suggesting superior efficacy of polybacterial formulations compared to single-strain interventions.

Despite the positive findings of this study, several limitations persist. First, inter-study heterogeneity was substantial, with I^2^ values of 72% for TNSS and 89% for IgE, potentially arising from strain diversity, dosage variations, inconsistent treatment durations, and baseline patient characteristics. Second, some included studies employed combined interventions where observed effects may reflect synergism rather than independent probiotic activity. Although random-effects models and subgroup analyses mitigated this issue, future research should adhere to the WHO Guidelines for the Design of Probiotic Clinical Trials, adopting a standardized intervention window, such as 12–16 week, while systematically reporting intervention seasons, regional pollen concentration data, and related parameters to enhance cross-study comparability. Furthermore, the utilization of endpoint values rather than change-from-baseline measurements, without proper adjustment for baseline characteristics, may introduce bias in estimating the true therapeutic effects of interventions. Moreover, most studies were short-term, lacking long-term follow-up data, thereby precluding assessment of the sustained effects of probiotics. Additionally, the samples were primarily derived from Asian and European populations, with an absence of data from African or Latin American regions, limiting the generalizability of the results. Finally, while SUCRA analysis facilitates strain ranking, it relies on indirect comparisons, and the efficacy of treatments requires further investigation.

In conclusion, this study demonstrates that probiotic interventions, particularly multi-strain, exhibit potential clinical value in pediatric allergic rhinitis, significantly improving symptoms and quality of life, while demonstrating nonsignificant effects on immunological parameters and clinical efficacy. Future research should prioritize large-scale, multicenter, long-term studies to explore optimal strain combinations, dosages, and mechanisms, incorporating diverse populations to further validate these findings and inform clinical practice.

## Data Availability

The original contributions presented in the study are included in the article/Supplementary Material, further inquiries can be directed to the corresponding authors.
