# Supplementary material for: Potential clinical benefits of probiotics in pediatric allergic rhinitis: a systematic review and network meta-analysis
Source: Front Pediatr. 2026 Mar 9;14:1744817. doi: 10.3389/fped.2026.1744817 (PMC13006500; doi:10.3389/fped.2026.1744817)
Supplement: Supplementary file 1 [file Supplementaryfile1.docx]

Supplementary Material

# Supplementary Figures and Tables

## Supplementary Figures


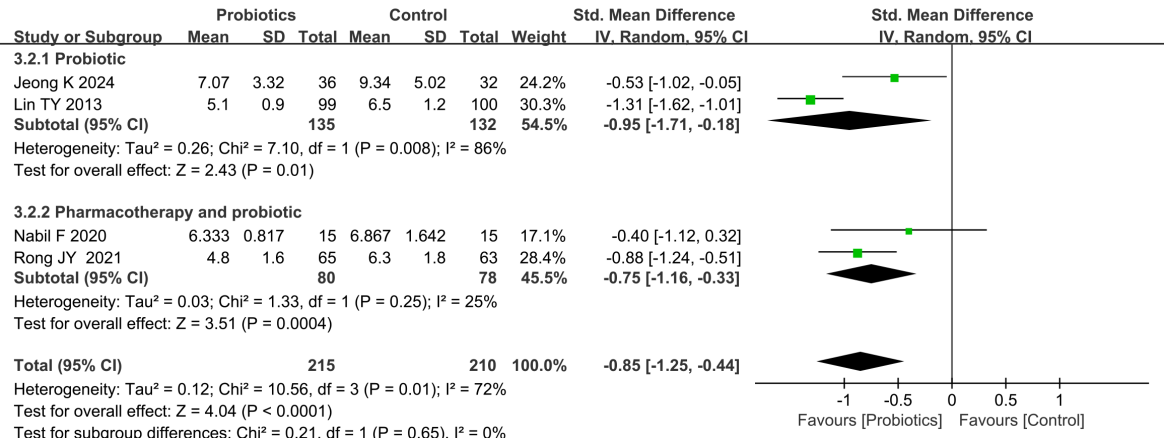


**Supplementary Figure 1.** Forest plot comparing the effect of probiotics and control on TNSS. Subgroup by the types of therapy.


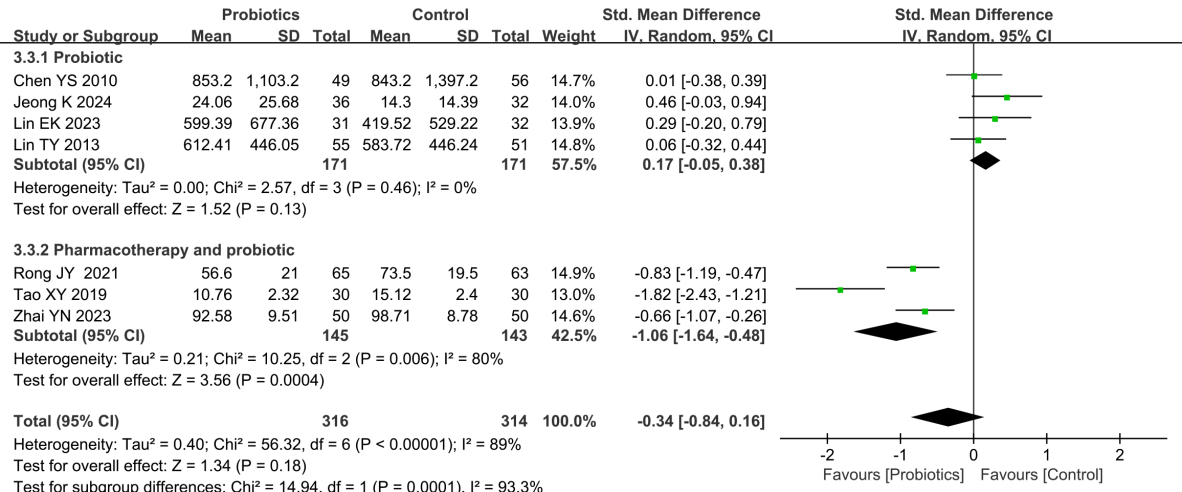


**Supplementary Figure 2.** Forest plot comparing the effect of probiotics and control on serum total IgE level. Subgroup by the types of therapy.


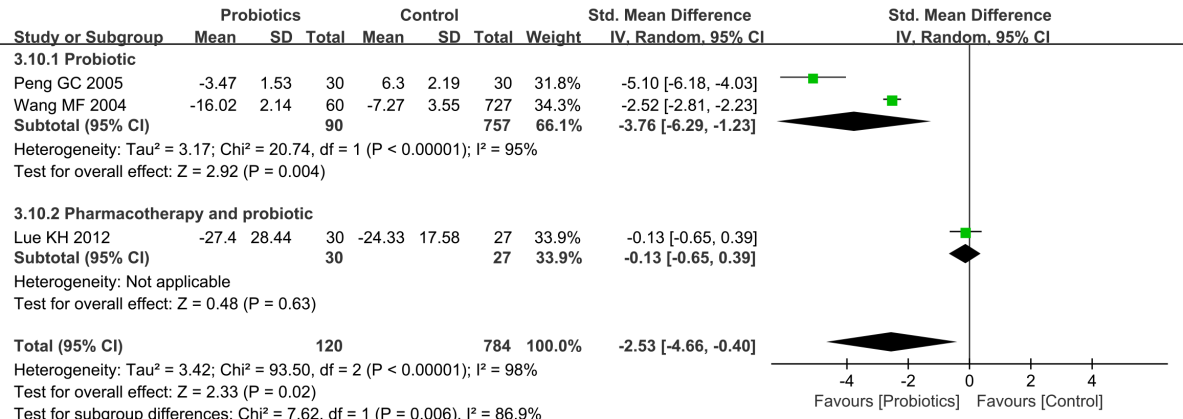


**Supplementary Figure 3.** Forest plot comparing the effect of probiotics and control on PRQLQ. Subgroup by the types of therapy.


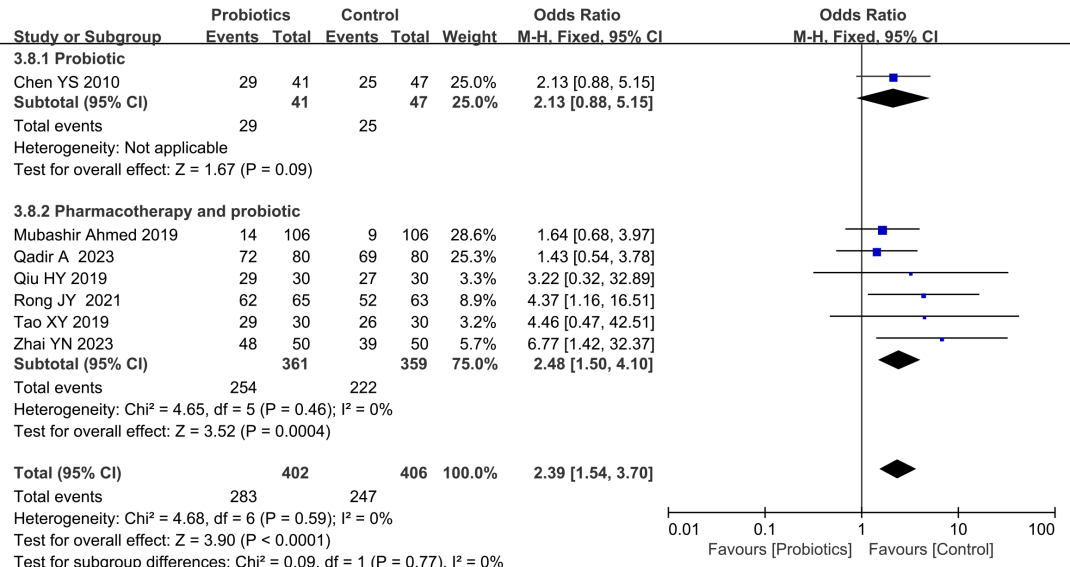


**Supplementary Figure 4.** Forest plot comparing the effect of probiotics and control on clinical efficacy. Subgroup by the types of therapy.


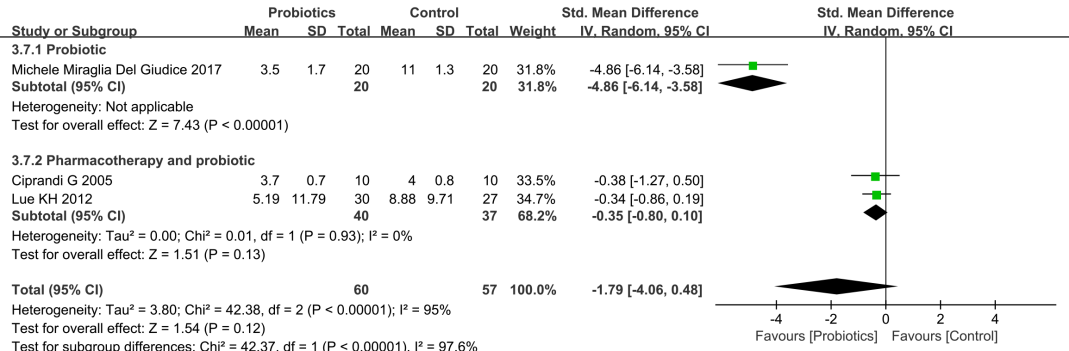


**Supplementary Figure 5.** Forest plot comparing the effect of probiotics and control on TSS. Subgroup by the types of therapy.

## Supplementary Tables

Table S1. Excluded Studies Inventory.

| Author Year | Age | Probiotics | Period | Control | Outcomes | Exclusion rationale |
| --- | --- | --- | --- | --- | --- | --- |
| Rong JY 2024 | 6-12 y | Bifidobacterium longum, L. acidophilus and Enterococcus faecalis | 6m | Conventional treatment: Environmental guidance, medication use based on condition, etc. | VAS, RQLQ | No available outcome measures |
| Liu XL 2023 | 5-7 y | Bifidobacterium infantis, L. acidophilus, Enterococcus faecalis, Bacillus cereus | 2 w | Mometasone furoate nasal spray | VAS, Serum IL-17, IL-10 | No available outcome measures |
| Jerzynska J 2016 | 5-12y | L. rhamnosusGG | 5m | SLIT-placebo | OSS, NSS, Immunological markers (e.g., IL-1, IL-6) | No available outcome measures |
| Rossberg S 2020 | 0-11y | Gram-negative Escherichia coli and Gram-positive Enterococcus faecalis | 5w-7m | Placebo | Prevalence of AR | No available outcome measures |
| West CE 2013 | 0-9y | L.paracasei | 8-9y | Placebo | Clinical symptom score | No available outcome measures |
| Giovannini M 2007 | 2-5y | 1. gasseri, treptococcus thermophilus, L. casei | 12m | Non-fermented milk | Clinical symptom assessment, IgE | Exclude food-based carriers such as fermented milk or cereals |
| Anania C 2021 | 6-14y | Bifidobacterium animalis, L. BB12, Enterococcus faecium L3 | 3m | Placebo (Maltodextrin) | NSS | No available outcome measures |
| Arthur C Ouwehand 2009 | 4-13y | L.acidophilus | 4m | Placebo (microcrystalline cellulose) | Specific IgE birch pollen, Blood eosinophils | No available outcome measures |
